# B3 Lesions at Vacuum-Assisted Breast Biopsy under Ultrasound or Mammography Guidance: A Single-Center Experience on 3634 Consecutive Biopsies

**DOI:** 10.3390/cancers13215443

**Published:** 2021-10-29

**Authors:** Veronica Girardi, Monica Guaragni, Nella Ruzzenenti, Fabrizio Palmieri, Gianluca Fogazzi, Andrea Cozzi, Diana Lucchini, Alberto Buffoli, Simone Schiaffino, Francesco Sardanelli

**Affiliations:** 1 Breast Radiology, EUSOMA-Certified Breast Unit, Istituto Clinico Sant’Anna, Via del Franzone 31, 25127 Brescia, Italy; veronica.girardi@grupposandonato.it; 2 Breast Pathology, EUSOMA-Certified Breast Unit, Istituto Clinico Sant’Anna, Via del Franzone 31, 25127 Brescia, Italy; monica.guaragni@grupposandonato.it (M.G.); nella.ruzzenenti@grupposandonato.it (N.R.); 3 Breast Surgery, EUSOMA-Certified Breast Unit, Istituto Clinico Sant’Anna, Via del Franzone 31, 25127 Brescia, Italy; fabrizio.palmieri@grupposandonato.it; 4 Breast Medical Oncology, EUSOMA-Certified Breast Unit, Istituto Clinico Sant’Anna, Via del Franzone 31, 25127 Brescia, Italy; gianluca.fogazzi@grupposandonato.it; 5 Department of Biomedical Sciences for Health, Università degli Studi di Milano, Via Mangiagalli 31, 20133 Milano, Italy; francesco.sardanelli@unimi.it; 6 Breast Psycho-Oncology, EUSOMA-Certified Breast Unit, Istituto Clinico Sant’Anna, Via del Franzone 31, 25127 Brescia, Italy; diana.lucchini@grupposandonato.it; 7 Radiation Oncology, EUSOMA-Certified Breast Unit, Istituto Clinico Sant’Anna, Via del Franzone 31, 25127 Brescia, Italy; alberto.buffoli@grupposandonato.it; 8 Unit of Radiology, IRCCS Policlinico San Donato, Via Morandi 30, 20097 San Donato Milanese, Italy; simone.schiaffino@grupposandonato.it

**Keywords:** breast neoplasms, high-risk lesions, B3 lesions, ultrasonography, mammography, image-guided biopsy, vacuum-assisted biopsy (VAB), underestimation, upgrade rate

## Abstract

**Simple Summary:**

Image-guided biopsy of suspicious findings at mammography or breast ultrasonography can result in the diagnosis of lesions with uncertain malignant potential (B3 lesions). These, in turn, can turn out to be cancer (i.e., they are upgraded) when larger specimens of tissue are examined after breast surgery, especially if these lesions belong to the B3b subcategory, characterized by a higher probability of malignancy than the B3a subcategory. This uncertain nature makes their management highly controversial. We aimed to report a particularly large series of B3 lesions—coming from an internationally certified Breast Unit—since such series are seldom available. In this series of 3634 consecutive biopsies, we found 604 B3 lesions, of which 17 (2.8%) were upgraded to malignancy after surgery. B3b lesions had an almost 12-fold higher upgrade rate (4.7%) than B3a lesions (0.4%), reinforcing the evidence that recommends surgery for B3b lesions and acknowledges the possibility of active surveillance of B3a lesions.

**Abstract:**

The rate of upgrade to cancer for breast lesions with uncertain malignant potential (B3 lesions) diagnosed at needle biopsy is highly influenced by several factors, but large series are seldom available. We retrospectively assessed the upgrade rates of a consecutive series of B3 lesions diagnosed at ultrasound- or mammography-guided vacuum-assisted biopsy (VAB) at an EUSOMA-certified Breast Unit over a 7-year timeframe. The upgrade rate was defined as the number of ductal carcinoma in situ (DCIS) or invasive cancer at pathology after excision or during follow-up divided by the total number of B3 lesions. All lesions were reviewed by one of four pathologists with a second opinion for discordant assessments of borderline cases. Excision or surveillance were defined by the multidisciplinary tumor board, with 6- and 12-month follow-up. Out of 3634 VABs (63% ultrasound-guided), 604 (17%) yielded a B3 lesion. After excision, 17/604 B3 lesions were finally upgraded to malignancy (2.8%, 95% confidence interval [CI] 1.8–4.5%), 10/17 (59%) being upgraded to DCIS and 7/17 (41%) to invasive carcinoma. No cases were upgraded during follow-up. B3a lesions showed a significantly lower upgrade rate (0.4%, 95% CI 0.1–2.1%) than B3b lesions (4.7%, 95% CI 2.9–7.5%, *p* = 0.001), that had a 22.0 adjusted odds ratio for upgrade (95% CI 2.1–232.3). No significant difference was found in upgrade rates according to imaging guidance or needle caliper. Surveillance-oriented management can be considered for B3a lesions, while surgical excision should be pursued for B3b lesions.

## 1. Introduction

Introduced in the early 1990s, image-guided needle biopsy has almost completely replaced surgical excision as the primary option for the histopathologic characterization of focal breast lesions [1,2,3]. The imaging modalities guiding percutaneous breast biopsies include ultrasound, X-ray (both digital mammography and tomosynthesis) and magnetic resonance imaging (MRI) [1]. Ultrasound guidance has many advantages over the other techniques, including a wider availability, lower costs, faster and real-time-guided procedures, and better patient comfort [1,4]. From the perspective of biopsy techniques, core needle biopsy has gradually replaced the use of fine-needle sampling (with selected exceptions) due to its superior lesion characterization and lower frequency of insufficient sampling [5,6]. Its last evolution, vacuum-assisted biopsy (VAB), has further refined the management of suspicious breast lesions [7,8,9].

One of the chief drawbacks of image-guided percutaneous biopsies is the diagnosis of lesions “with uncertain malignant potential”, also known as “B3” lesions, which are associated with a wide spectrum of upgrade rates, i.e., a subsequent diagnosis of cancer at final pathology after surgical excision or during follow-up [10,11,12,13]. The key issue is that the management of these lesions is highly controversial and is therefore the center of a lively debate, that could be fittingly approached by building evidence from large series, which are still sporadically available [14,15,16]. If performed with large-caliber needles, VAB seems to consistently improve lesion characterization in B3 lesions [17,18,19,20,21,22,23,24], even assuming a therapeutic role with vacuum-assisted excision, which is increasingly being proposed with interesting results [25,26,27,28].

The aim of this study is to present a large retrospective single-center experience with consecutive B3 lesions diagnosed at ultrasound- or mammography-guided VAB over a 7-year timeframe, analyzing the distributions of B3 lesion subtypes and their upgrade rates at surgical excision or during follow-up.

## 2. Materials and Methods

This study was approved by the local Ethics Committee (Comitato Etico di Brescia, control number NP 4789) on 17 June 2021. The need for patients’ informed consent was waived due to the retrospective nature of the study. Consecutive VAB procedures performed under ultrasound or stereotactic X-ray guidance at a single center (Istituto Clinico Sant’Anna, Brescia, Italy) from January 2011 to June 2018 were reviewed, including only B3 diagnoses, with an available final pathologic diagnosis at surgical excision or imaging follow-up of at least 24 months. All B3 lesions diagnosed according to the “World Health Organization classification of tumors of the breast—2019” [29] were included.

During the study period, an ultrasound system and a mammography unit equipped with stereotaxis were available to guide percutaneous biopsies. All VAB procedures were performed by one of two experienced breast radiologists (one with over 7000 mammograms read per year, 13 years of breast ultrasound experience and over 300 VABs per year in the last 10 years; the other, with over 5000 mammography per year, 13 years of breast ultrasound experience, and over 300 VABs per year in the last 10 years).

### 2.1. Ultrasound-Guided Vacuum-Assisted Biopsy

Ultrasound guidance was the first choice for percutaneous biopsy of ultrasound-detected breast lesions and of lesions detected by mammography or MRI that had corresponding findings at the targeted ultrasound examination. Radiologists used an ultrasound system with a 4–15 MHz high-resolution linear array transducer (MyLab™ Six, Esaote, Genova, Italy) and a 14-gauge vacuum suction device (FINESSE^®^ ULTRA Breast Biopsy System, Bard Biopsy, Tempe, AZ, USA), with real-time needle tracking. Post-fire needle position check was performed in all cases. The number of collected specimens in each procedure was eight or more per lesion, depending on each operator’s personal choice, target lesion dimensions, or potential incorrect post-fire needle position. For lesions thought to be difficult to visualize at subsequent ultrasound examinations, such as small lesions or mammography-detected lesions, a marker (localizing clip) was placed in the biopsy site after the procedure. The clip (Gel Mark UltraCor™ Breast Tissue Marker, Bard Biopsy, Tempe, AZ, USA) was placed through a coaxial cannula following the same path as the biopsy needle. For mammography-detected lesions, the correct position of the clip in the target lesion was checked with a post-biopsy mammogram. When the ultrasound-guided VAB was performed in the presence of calcifications, a mammogram of the specimens was performed to confirm the presence of calcifications within the samples.

### 2.2. Mammography-Guided Vacuum-Assisted Biopsy

Different types of biopsy systems for mammography-guided VAB were used in the study period. All procedures were performed using a stereotactic guidance (Mammomat Inspiration, Siemens Healthineers, Erlangen, Germany). A single-unit breast biopsy console with a hand piece (EnCor Enspire™ Breast Biopsy System, Bard Biopsy, AZ, USA) was used throughout the study period, with a 10-gauge vacuum-assisted probe (EnCor Biopsy Probe 10-gauge, Bard Biopsy, Tempe, AZ, USA) being used in all procedures before May 2013. From June 2013 onwards, a 7-gauge probe (EnCor Biopsy Probe 7-gauge, Bard Biopsy, Tempe, AZ, USA) was also available, and became the first choice in all procedures: the 10-gauge probe was then used only when biopsy with the 7-gauge probe was deemed unfeasible by the biopsy system software due to the lesion position and breast thickness. The number of collected specimens in each procedure was 12 or more per lesion, depending on each operator’s personal choice, target lesion dimensions, or potential incorrect post-fire needle position. Mammographic examination for the ascertainment of the clip positioning and specimen mammography (to separate samples with and without calcifications) were performed after all mammography-guided VABs.

### 2.3. Pathology after Needle Biopsy

Four pathologists (one with >30-year experience in breast pathology and the other three with at least 10 years of experience in breast pathology) were involved in the assessment of percutaneous specimens during the study period. A second-opinion strategy was used for borderline cases, such as differential diagnosis between atypical hyperplasia and ductal carcinoma in situ (DCIS), in cases with minimal findings of atypia, and in case of discrepancy between percutaneous biopsy and surgical specimen diagnoses. In case of discordant opinions, consensus between the two pathologists was reached through case-based discussion.

B3 lesions were classified into the following categories according to the World Health Organization classification [29]: atypical intraductal epithelial proliferation including atypical ductal hyperplasia (ADH); columnar cell hyperplasia (CCH) with atypia; flat epithelial atypia (FEA); lobular neoplasia, including atypical lobular hyperplasia (LIN1) and classical lobular carcinoma in situ (LIN2); radial scar or complex sclerosing lesion (RS-CSL); papillary lesions (PL); phyllodes tumor (PT); mucocele-like lesions (MLS); other unclassified or not otherwise specified B3 lesions, including lesions that could not be categorized into any of the previous categories.

Furthermore, all B3 lesions were classified into two subcategories (B3a or B3b), according to the absence or presence of cytologic atypia, respectively [30,31,32]: no specific cytologic atypia criteria were used other than the standard pathological criteria [33,34,35].

### 2.4. Radiologic–Pathologic Correlation and Management

Concordance between the pathology report and imaging findings was assessed by the radiologist for each case, while management recommendations were provided by the multidisciplinary tumor board by consensus. Each case underwent multidisciplinary discussion, referring women either to imaging follow-up or to surgical excision. In the case of B3a lesions, patients underwent surgery if they were symptomatic, if surgery was recommended by the referring physician or if they expressed a clear preference for surgical removal of the B3 lesion. Conversely, in case of B3b lesions, follow-up was proposed only for small lesions with complete removal after VAB in patients without increased breast cancer risk. Imaging follow-up (digital mammography and/or ultrasound, according to the image guidance of the percutaneous biopsy and to the clinical setting) was performed at 6 months and then annually with a joint clinical examination. Surgical excision was suggested in the following cases: presence of clinically suspicious lesion features, such as palpability or spontaneous secretion from a single ductal orifice; high radiological suspicion, such as a solid mass with microcalcifications or architectural distortion; absence of radiologic–pathologic correlation; direct request by the patient or referring physician; presence of coexisting risk factors such as previous malignancy or high-risk breast disease, family history of breast cancer in first-degree relatives, previous radiation treatment to the chest.

### 2.5. Surgical Excision, Surgical Pathology, and Follow-Up

Surgical excision was performed after ultrasound or mammographic localization with wire guide, usually within 4–8 weeks. The guide wire tip was positioned in the residual lesion, in the post-VAB hematoma in case of complete removal of the target lesion, and near the localizing non-migrated clip in the remaining cases.

Imaging check of the surgical specimen (with mammography or ultrasonography, as appropriate according to EUSOMA criteria) was directly performed during the surgical time, to verify the complete inclusion of the target lesion in the excised sample. In case of incomplete resection of the target lesion, an enlargement of surgical excision was immediately carried out after discussion with the surgeon.

The same four pathologists that classified percutaneous biopsies were also tasked to classify histology findings at surgical excision, either as malignant or benign lesions according to El-Sayed et al. [33] and de Beça et al. [34]. B3 cases were considered “upgraded” only if malignancy (i.e., invasive carcinoma or DCIS) was reported at pathology after surgical excision or during follow-up. All other B3 lesions (including those with atypical proliferative findings at surgical pathology) were considered as “not upgraded”. Finally, a retrospective review of all cases sent to follow-up was carried out to identify the performance of subsequent biopsies prompted by other findings. For cases referred for surgery, information on concomitant surgery in the ipsilateral or contralateral breast for synchronous B5 lesions was retrieved.

### 2.6. Statistical Analysis

Upgrade rates were calculated with their 95% confidence intervals (95% CIs), both overall and by lesions’ subgroups according to B3 types, atypia presence (B3a versus B3b), type of image guidance, and needle caliper. Direct and stratified comparisons of upgrade rates among subgroups were conducted with the Fisher’s exact test and the Mantel–Haenszel test, respectively. Analyses were performed using SPSS v.26.0 (IBM SPSS Inc., Chicago, IL, USA).

## 3. Results

In the study timeframe, 83,269 mammograms (19,617 (24%) as biennial examinations in the population-based organized screening program and 63,652 (76%) as spontaneous referral by symptomatic or asymptomatic women) and 56,752 breast ultrasound examinations were performed. The work-up process after recall for suspicious findings led to 3634 image-guided VABs (Table 1), performed under ultrasound guidance in 2280/3634 cases (63%) and under stereotactic guidance in 1354/3634 cases (37%). 

As shown in Table 2, pathology examination of the biopsy specimens found a total of 604/3634 (17%) B3 lesions. Of these, 246/604 (40.7%) were biopsied under ultrasound guidance, the remaining 358 (59.3%) under stereotactic guidance. A total of 262/604 (43.4%) B3 lesions were classified in the B3a category, the remaining 342 (56.6%) were classified in the B3b category. B3a lesions were far more commonly found among the 246 ultrasound-guided VABs performed with 14-gauge needles (171/246, 69.5%, 95% CI 63.5–74.9%) than among the 358 VABs performed under stereotactic guidance (91/358, 25.4%, 95% CI 21.2–30.2%, *p* < 0.001) with larger needle calipers (7-gauge and 10-gauge). A total of 355/604 B3 lesions (58.8%) were referred for surgery; as expected, this occurred more frequently in the B3b category (240/342, 70.2%, 95% CI 65.1–74.8%) than in the B3a category (115/262, 43.9%, 95% CI 38.0–49.9%, *p* < 0.001). Among the 240 B3b lesions originally referred for surgery, we found 8 cases (3.3%, 4 ADHs, 3 FEAs, 1 LIN2) in which concomitant surgery for synchronous B5 lesions was performed in another quadrant of the ipsilateral breast (3 cases) or in the contralateral breast.

Overall, 17/604 B3 lesions (2.8%) were upgraded to malignant ones, all of them at final pathology on surgical specimens: 10/17 (59%) as DCIS and 7/17 (41%) as invasive carcinoma (4 of ductal and 3 of lobular type), for an overall upgrade rate of 2.8% (95% CI 1.8–4.5%). None of the 249 cases sent to follow-up were upgraded during the analyzed timeframe, while 2/249 (0.8%) cases had a subsequent biopsy prompted by other findings detected during follow-up examinations and were referred for surgery. The first was a 51-year-old woman sent to follow-up with a diagnosis of ADH, who had a biopsy-proven diagnosis of DCIS (G2) in another quadrant of the same breast 5 years later. The second was a 71-year-old woman sent to follow-up with a PL of the right breast in September 2018, who also had a B5 lesion in the left breast that was surgically removed a month later (with a diagnosis of invasive mucinous carcinoma on the surgical specimen). 

Among B3 subtypes, LIN2 showed the highest upgrade rate (16.0%, 95% CI 6.4–34.7%), followed by ADH (5.2%, 95% CI 2.7–9.9%), LIN1 (2.5%, 95% CI 0.4–12.9%), FEA (2.4%, 95% CI 0.8–6.9%), and PLs (1.1%, 95% CI 0.2–6.0%), with a *p* = 0.012 at Fisher’s exact test for multiple comparison. B3a lesions showed an overall upgrade rate of 0.4% (95% CI 0.1–2.1%), while B3b lesions had an overall upgrade rate of 4.7% (95% CI 2.9–7.5%, *p* = 0.001), with an unadjusted 12.8 odds ratio for upgrade (95% CI 1.7–97.2) and an adjusted 22.0 common odds ratio for upgrade (95% CI 2.1–232.3, Mantel–Haenszel test of conditional independence *p* = 0.003), after controlling for image guidance.

As shown in Table 3, no significant difference was found between the overall upgrade rates of B3 lesions diagnosed with ultrasound-guided VABs (2.4%, 95% CI 1.1–5.2%) and mammography-guided VABs (3.1%, 95% CI 1.7–5.4%, *p* = 0.804). When subgrouping B3 lesions according to presence of atypia, B3b lesions were significantly more frequently detected at mammography-guided VAB (267/358, 74.6%, 95% CI 69.8–78.8%) than at ultrasound-guided VAB (75/246, 30.5%, 95% CI 25.1–36.5%, *p* < 0.001). A significant difference was found between upgrade rates of the 171 B3a lesions (upgrade rate 1/171, 0.6%, 95% CI 0.1–3.2%) and the 75 B3b lesions (upgrade rate 5/75, 6.7%, 95% CI 2.9–14.7%, *p* = 0.011) diagnosed with ultrasound-guided VAB, while no significant difference in upgrade rates was found between the 91 B3a lesions (none of them upgraded, 0%) and the 267 B3b lesions diagnosed with mammography-guided VAB (upgrade rate 11/267, 4.1%, 95% CI 2.3–7.2%, *p* = 1.000).

Further subgroup comparisons of upgrade rates according to needle caliper (Table 4) was partially hindered by substantial overlap with image guidance categories, since different needle calipers were used only among mammography-guided VABs, with 133 out of 358 (37.2%) being performed with a 7-gauge needle, the remaining 225/358 (62.8%) with a 10-gauge needle, while all 246 ultrasound-guided VABs were performed with a 14-gauge needle. Indeed, no significant difference was found among upgrade rates of B3 subtypes stratified by needle caliper, nor by more specific comparison of B3a and B3b lesions upgrade rates in the 7-gauge (*p* = 0.572) and in the 10-gauge subgroup (*p* = 0.194) at mammography-guided VAB.

## 4. Discussion

The controversial management of B3 lesions has been discussed in several guidelines, such as those issued by the American Society of Breast Surgeons [36], by the UK National Health Service [11], and in the two statements drafted by the European-centered International Consensus Conferences of 2016 [37] and 2018 [14]. A general agreement can only be found for the management of phyllodes tumor, for which surgical excision is always recommended, while potential approaches for all the remaining B3 lesions are surgical excision, vacuum-assisted excision (VAE), or follow-up [38,39]. B3 lesions with atypia are generally managed with an intervention-oriented approach: as explained in the guidelines issued by the UK National Health Service, this choice tends to allow a more precise characterization of the extent of the atypia around the 3 mm diagnostic cut-off between atypical hyperplasia and DCIS, albeit with the obvious disadvantages of patients’ discomfort brought about by the invasive procedure and the related potential complications. Surgical excision is generally recommended in the USA, while European guidelines tend to favor VAE, albeit with some discrepancies between UK recommendations and those of the International Consensus Conferences [15,16,40]. The role of imaging surveillance—which is sometimes proposed even for B3b lesions—is the chief topic of an extensive debate [38,39] that, like the widespread lack of consensus, stems from the high variability in the rates of upgrade to malignancy [16,38,39]. Proponents of the surveillance-based approach draw on the actual relatively low grade of treatment personalization and advocate various strategies (such as those implemented in EUSOMA-certified centers) to curtail invasive procedures without engendering underestimation of the subsequent patient-specific risk of developing breast cancer, while opponents point out the absence of standardized protocols and of substantial evidence in favor of extending this approach to a constantly-higher number of B3 subtypes [10,15,40]. Generally speaking, while further work-up with contrast-enhanced imaging [41,42,43,44], predictive models [45,46], and artificial intelligence tools [47] yielded promising results, none of these approaches has been established as a reliable guide for the management of B3 lesions.

In this study, focused on B3 lesions detected at ultrasound- and stereotactic-guided VAB, we present one of the largest available series for these lesions, from an EUSOMA-certified [48] Breast Unit, with a double-reading pathology reporting approach for borderline cases that was constantly implemented in the last 7 years. Among the 604 B3 lesions (17% of the 3634 VAB performed in the study period), we found a low overall upgrade rate of 2.8%, which was completely driven by lesions referred for surgery (no cases were upgraded during follow-up) and strongly influenced by lesion subtype, with B3b lesions (4.7% upgrade rate) having an adjusted 22.0 odds ratio for upgrade compared to B3a lesions (0.4% upgrade rate). While lower than those reported by the recent literature on both B3a and B3b lesions [13,49,50,51,52], these upgrade rates match the general trend that sees lesions with atypia (i.e., B3b lesions such as ADH or LIN) having higher upgrade rates than those of lesions without atypia (i.e., B3a lesions such as RS-CSL and PLs). Conversely, no difference was found between upgrade rates according to guidance or needle caliper, as already postulated by Philpotts et al. [53] in 2003, when comparing the use of 14-gauge automated biopsy devices and 11-gauge VAB devices in terms of missed lesions, underestimation, complications, or the need (immediate or delayed) for a second biopsy.

From a clinical perspective, our study further confirms, in a large series, the results of recent meta-analyses [13,49,50,52,54] that demonstrated how upgrade rates for B3a lesions are well below the 2% threshold, which, according to the Breast Imaging Reporting and Data System, would justify the adoption of a surveillance-centered option [55], while the same values for B3b lesions (5.2% for ADH and 2.5% for FEA in our series) are still far above this threshold. While the 2% threshold and the intervention-oriented perspective were strongly challenged by the Second International Consensus Conference in 2018 [14,16] and by another recent meta-analysis on LIN [51], a sudden change in management could be still delayed by robust assistance strategies to reduce overtreatment [56]. These strategies include a second-opinion assessment of biopsy specimens [57] (as implemented in our center for borderline cases) or the application of artificial intelligence for upgrade prediction [47] and could gain widespread application in the future. Another less invasive but still viable approach, particularly in light of recent results, could be the careful integration of VAE with surveillance, provided that the radiologic–pathological correlation is performed and that the removed tissue shows a low presence of atypical cytological features [16,24,25,26,27,28]. Of note, however, the need for personalized imaging surveillance (not limited to biennial mammography but potentially including supplemental ultrasound, digital breast tomosynthesis, contrast-enhanced MRI, and contrast-enhanced mammography) could be postulated for all patients with B3 lesions, also after surgical treatment. The notion—previously hinted by other studies [15]—that the presence of any B3 lesion in the breast increases the lifetime risk of ipsilateral or contralateral breast cancer (albeit at different degrees) seems to concur with the fact that 10 patients of our series (1.7%, eight of them with a referral to surgery) had a synchronous or subsequent diagnosis of invasive carcinoma in another quadrant of the ipsilateral breast or in the contralateral breast.

The first limitation of this study is its retrospective design, even though the clinical context of the management of B3 lesions makes this limitation a minor one, since the inclusion of one of the largest available consecutive series should compensate for potential biases. More importantly, some concerns regarding the generalizability of our results could be paradoxically represented by the high experience of the two radiologists and the four pathologists, all of them working at the certified Breast Unit, where all cases were gathered. In other words, the specific sub-specialization of the operators involved in this study may limit the generalizability of these results to those centers with similar characteristics and probably explains the lower upgrade rates found in our series compared to those reported by recent syntheses of the available literature.

## 5. Conclusions

Our study showed how VAB, performed under ultrasound or stereotactic guidance at a certified Breast Unit with highly experienced radiologists and pathologists, offers great potential to improve diagnostic accuracy in B3 lesions regardless of needle caliper, as well as reducing the need for subsequent open biopsy, containing the overall upgrade rate of around 3%. While all B3 lesions subtypes without atypia had an upgrade rate that could prompt a more conservative approach, upgrade rates for B3 lesions with atypia remained well above the 2% threshold, justifying surveillance over surgical excision.

## Figures and Tables

**Table 1:** Vacuum-assisted biopsies performed from January 2011 to June 2018.

Type of Guidance	Number of VABs	Pathologic Category on VAB Samples			
		B2	B3	B4	B5
Ultrasound	2280 (62.7%)	1403 (38.6%)	246 (6.8%)	7 (0.2%)	624 (17.1%)
Mammography	1354 (37.3%)	722 (19.9%)	358 (9.8%)	6 (0.2%)	268 (7.4%)
**Total**	**3634 (100.0%)**	**2125 (58.5%)**	**604 (16.6%)**	**13 (0.4%)**	**892 (24.5%)**

VAB: vacuum-assisted biopsy.

**Table 2:** Subtypes, management, and upgrade rates of the 604 included B3 lesions.

Lesion			Surgery		Outcome				Upgrade	
Type	Subtype	Total	Yes	No	Malignant		Benign	Negative at FU	Upgraded	Upgrade Rate (95% CI)
					Invasive	DCIS				
**B3a**	PL	91	47	44	1	0	46	44	**1**	1.1% (0.2–6.0%)
	PT	62	40	22	0	0	40	22	0	0.0%
	RS-CSL	79	25	54	0	0	25	54	0	0.0%
	CCH	24	2	22	0	0	2	22	0	0.0%
	MLS	6	1	5	0	0	1	5	0	0.0%
**Subtotal B3a**		**262**	**115**	**147**	**1**	**0**	**114**	**147**	**1**	**0.4% (0.1–2.1%)**
**B3b**	ADH	154	125	29	3	5	117	29	8	5.2% (2.7–9.9%)
	FEA	123	62	61	0	3	59	61	3	2.4% (0.8–6.9%)
	LIN1	40	28	12	0	1	27	12	1	2.5% (0.4–12.9%)
	LIN2	25	25	0	3	1	21	0	4	16.0% (6.4–34.7%)
**Subtotal B3b**		**342**	**240**	**102**	**6**	**10**	**224**	**102**	**16**	**4.7% (2.9–7.5%)**
**Grand total**		**604**	**355**	**249**	**7**	**10**	**338**	**249**	**17**	**2.8% (1.8–4.5%)**

DCIS: ductal carcinoma in situ; FU: follow-up; CI: confidence interval; PL: papillary lesions; PT: phyllodes tumor; RS-CSL: radial scar or complex sclerosing lesion; CCH: columnar cell hyperplasia with atypia; MLS: mucocele-like lesion; ADH: atypical ductal hyperplasia; FEA: flat epithelial atypia; LIN 1: atypical lobular hyperplasia; LIN2: lobular carcinoma in situ.

**Table 3:** Biopsy guidance-oriented analysis of the 604 B3 lesions according to surgery, outcome, and upgrade rates.

	Lesion			Surgery		Outcome				Upgrade	
Guidance	Type	Subtype	Total	Yes	No	Malignant		Benign	Negative at FU	Upgraded	Upgrade Rate (95% CI)
						Invasive	DCIS				
**US-guided**	**B3a**	PL	58	38	20	1	0	37	20	1	1.7% (0.3–9.1%)
		PT	62	40	22	0	0	40	22	0	0.0%
		RS-CSL	42	20	22	0	0	20	22	0	0.0%
		CCH	9	2	7	0	0	2	7	0	0.0%
		MLS	0	0	0	0	0	0	0	0	0.0%
	**Subtotal B3a**		**171**	**100**	**71**	**1**	**0**	**99**	**71**	**1**	**0.6% (0.1–3.2%)**
	**B3b**	ADH	35	33	2	0	3	30	2	3	8.6% (3.0–22.4%)
		FEA	19	14	5	0	0	14	5	0	0.0%
		LIN1	15	10	5	0	1	9	5	1	6.7% (1.2–29.8%)
		LIN2	6	6	0	1	0	5	0	1	16.7% (3.0–56.4%)
	**Subtotal B3b**		**75**	**63**	**12**	**1**	**4**	**58**	**12**	**5**	**6.7% (2.9–14.7%)**
**Total US**			**246**	**163**	**83**	**2**	**4**	**157**	**83**	**6**	**2.4% (1.1–5.2%)**
**MX-guided**	**B3a**	PL	33	9	24	0	0	9	24	0	0.0%
		PT	0	0	0	0	0	0	0	0	0.0%
		RS-CSL	37	5	32	0	0	5	32	0	0.0%
		CCH	15	0	15	0	0	0	15	0	0.0%
		MLS	6	1	5	0	0	1	5	0	0.0%
	**Subtotal B3a**		**91**	**15**	**76**	**0**	**0**	**15**	**76**	**0**	**0.0%**
	**B3b**	ADH	119	92	27	3	2	87	27	5	4.2% (1.8–9.5%)
		FEA	104	48	56	0	3	45	56	3	2.9% (1.0–8.1%)
		LIN1	25	18	7	0	0	18	7	0	0.0%
		LIN2	19	19	0	2	1	16	0	3	15.8% (5.5–37.6%)
	**Subtotal B3b**		**267**	**177**	**90**	**5**	**6**	**166**	**90**	**11**	**4.1% (2.3–7.2%)**
**Total MX**			**358**	**192**	**166**	**5**	**6**	**181**	**166**	**11**	**3.1% (1.7–5.4%)**

US: ultrasound; MX: mammography; DCIS: ductal carcinoma in situ; FU: follow-up; CI: confidence interval; PL: papillary lesions; PT: phyllodes tumor; RS-CSL: radial scar or complex sclerosing lesion; CCH: columnar cell hyperplasia with atypia; MLS: mucocele-like lesion; ADH: atypical ductal hyperplasia; FEA: flat epithelial atypia; LIN 1: atypical lobular hyperplasia; LIN2: lobular carcinoma in situ.

**Table 4:** Needle caliper subgroups for the 358 B3 lesions that underwent stereotactic-guided vacuum-assisted biopsy.

	Lesion			Surgery		Outcome				Upgrade	
Needle Caliper	Type	Subtype	Total	Yes	No	Malignant		Benign	Negative at FU	Upgraded	Upgrade Rate (95% CI)
						Invasive	DCIS				
**7-gauge**	**B3a**	PL	14	3	11	0	0	3	11	0	0%
		PT	0	0	0	0	0	0	0	0	0%
		RS-CSL	9	2	7	0	0	2	7	0	0%
		CCH	6	0	6	0	0	0	6	0	0%
		MLS	3	0	3	0	0	0	3	0	0%
	**Subtotal B3a**		**32**	**5**	**27**	**0**	**0**	**5**	**27**	**0**	0%
	**B3b**	ADH	44	33	11	1	0	32	11	1	**2.3% (0.4–11.8%)**
		FEA	36	19	17	0	2	17	17	2	**5.6% (1.5–18.1%)**
		LIN1	13	7	6	0	0	7	6	0	0%
		LIN2	8	8	0	0	1	7	0	1	**12.5% (2.2–47.1%)**
	**Subtotal B3b**		**101**	**67**	**34**	**1**	**3**	**63**	**34**	**4**	**4.0% (1.6–9.7%)**
**Total 7-gauge**			**133**	**72**	**61**	**1**	**3**	**68**	**61**	**4**	**3.0% (1.2–7.5%)**
**10-gauge**	**B3a**	PL	19	6	13	0	0	6	13	0	0%
		PT	0	0	0	0	0	0	0	0	0%
		RS-CSL	28	3	25	0	0	3	25	0	0%
		CCH	9	0	9	0	0	0	9	0	0%
		MLS	3	1	2	0	0	1	2	0	0%
	**Subtotal B3a**		**59**	**10**	**49**	**0**	**0**	**10**	**49**	**0**	0%
	**B3b**	ADH	75	59	16	2	2	55	16	4	**5.3% (2.1–12.9%)**
		FEA	68	29	39	0	1	28	39	1	**1.5% (0.3–7.9%)**
		LIN1	12	11	1	0	0	11	1	0	0%
		LIN2	11	11	0	2	0	9	0	2	**18.2% (5.1–47.7%)**
	**Subtotal B3b**		**166**	**110**	**56**	**4**	**3**	**103**	**56**	**7**	**4.2% (2.1–8.4%)**
**Total 10-gauge**			**225**	**120**	**105**	**4**	**3**	**113**	**105**	**7**	**3.1% (1.5–6.3%)**

DCIS: ductal carcinoma in situ; FU: follow-up; CI: confidence interval; PL: papillary lesions; PT: phyllodes tumor; RS-CSL: radial scar or complex sclerosing lesion; CCH: columnar cell hyperplasia with atypia; MLS: mucocele-like lesion; ADH: atypical ductal hyperplasia; FEA: flat epithelial atypia; LIN 1: atypical lobular hyperplasia; LIN2: lobular carcinoma in situ.

## Data Availability

All data collected for this study are presented in this paper.
